# Targeted and Reversible Blood-Retinal Barrier Disruption via Focused Ultrasound and Microbubbles

**DOI:** 10.1371/journal.pone.0042754

**Published:** 2012-08-13

**Authors:** Juyoung Park, Yongzhi Zhang, Natalia Vykhodtseva, James D. Akula, Nathan J. McDannold

**Affiliations:** 1 Department of Radiology, Brigham and Women's Hospital and Harvard Medical School, Boston, Massachusetts, United States of America; 2 Department of Ophthalmology, Children's Hospital Boston and Harvard Medical School, Boston, Massachusetts, United States of America; University of Regensburg, Germany

## Abstract

The blood-retinal barrier (BRB) prevents most systemically-administered drugs from reaching the retina. This study investigated whether burst ultrasound applied with a circulating microbubble agent can disrupt the BRB, providing a noninvasive method for the targeted delivery of systemically administered drugs to the retina. To demonstrate the efficacy and reversibility of such a procedure, five overlapping targets around the optic nerve head were sonicated through the cornea and lens in 20 healthy male Sprague-Dawley rats using a 690 kHz focused ultrasound transducer. For BRB disruption, 10 ms bursts were applied at 1 Hz for 60 s with different peak rarefactional pressure amplitudes (0.81, 0.88 and 1.1 MPa). Each sonication was combined with an IV injection of a microbubble ultrasound contrast agent (Definity). To evaluate BRB disruption, an MRI contrast agent (Magnevist) was injected IV immediately after the last sonication, and serial T1-weighted MR images were acquired up to 30 minutes. MRI contrast enhancement into the vitreous humor near targeted area was observed for all tested pressure amplitudes, with more signal enhancement evident at the highest pressure amplitude. At 0.81 MPa, BRB disruption was not detected 3 h *post* sonication, after an additional MRI contrast injection. A day after sonication, the eyes were processed for histology of the retina. At the two lower exposure levels (0.81 and 0.88 MPa), most of the sonicated regions were indistinguishable from the control eyes, although a few tiny clusters of extravasated erythrocytes (petechaie) were observed. More severe retinal damage was observed at 1.1 MPa. These results demonstrate that focused ultrasound and microbubbles can offer a noninvasive and targeted means to transiently disrupt the BRB for ocular drug delivery.

## Introduction

Delivering pharmaceutical agents to specific retinal locations may greatly improve treatment of degenerative retinopathies, including age-related macular degeneration, diabetic retinopathy, and hereditary retinal disorders such as Norrie disease. However, delivery of most therapeutic agents to the retina from the peripheral circulation is limited by the membrane impermeability of blood-retinal barrier (BRB) [1], which is formed by complex tight junctions of the endothelium of retinal blood vessels and the retinal pigment epithelium [2]. The BRB also presents a functional impediment for drug delivery due to efflux transporters such as P-glycoprotein [3] that rapidly eliminate many substances from the extravascular space. Currently an estimated 98% of clinically validated drugs, of which many are potential therapies for retinal diseases, cannot cross the BRB [4], [5], [6], [7].

Multiple methods, including topical, systemic, periocular, and intravitreal approaches, are currently used to deliver drugs to the eye [8]. If the retina is the target, topical, systemic and periocular approaches are limited due to the presence of the BRB and other ocular barriers. Intravitreal injections can effectively deliver drugs to the fundus, but this is an unpleasant, invasive procedure with risk of retinal detachment, retinal hemorrhage, endophthalmitis and glaucoma [9], [10]. For chronic diseases requiring repeated intraocular injections, the risks multiply. In some cases, intravitreal approaches may also not be able to deliver sufficient drug to the retina due to the inner limiting membrane (the border between the vitreous humor and the retina) [11]. Hence, the development of minimally-invasive and efficient techniques to bypass the BRB and allow passage of otherwise non-permeable therapeutic agents to the retinal tissue is desired.

A decade ago, a noninvasive, targeted, and reversible technique that combines low-energy ultrasound bursts with IV-administered microbubble ultrasound contrast agent to temporarily induce blood-brain barrier (BBB) disruption was found [12]. The technique has been shown to induce blood-brain barrier disruption without other significant side effects [13], [14], [15], to deliver even large-molecular drugs [16], and to improve therapeutic outcomes in animal disease models [17], [18]. This technique can also increase delivery of anti-cancer agents to tumors which have pathological blood vessels that lack a fully-intact BBB [17]. Importantly, the barrier is restored a few hours after sonication [12], [19], [20], providing a time window sufficiently long for drug delivery but short enough to escape the toxic effects of long-term BBB disruption.

The mechanisms that result in BBB disruption using this technique have not been fully elucidated. It does not appear to be the result of violent microbubble collapse (i.e., inertial cavitation) [15] or bulk tissue heating [12]. Histologic and electron microscopic data indicate that the disruption is not caused by permanent vessel damage (i.e. tears or rupture), but instead it appears to be caused by the mechanical stimulation that induces a temporary widening of tight junctions [20] and results in active transport [21] that allows drugs to be delivered to the brain. There are several microbubble dynamics activated by an ultrasound pressure field that will create forces on the vasculature, including acoustic radiation force on the microbubbles, bubble oscillation, and acoustic streaming [21]. These forces may induce direct physical changes to the endothelium, or they could potentially evoke biological changes such as calcium concentration changes [22], [23] or Akt activation [24], which could induce the BBB disruption. Ultrasound and microbubble interactions can also generate transient pores in cell membranes (i.e, sonoporation), a process thought to be due to shear stress induced by acoustic streaming [25] or other forces induced the microbubble dynamics. Whether such pores are created in the endothelial cells during sonications that produce BBB disruption, and their role in the disruption process are not known.

The retina and several other organs of the body also have vascular barriers similar to the BBB that restrict passage of systemically-administered substances. The mechanical stimulation on the blood vessels provided by the ultrasound exposures and the microbubbles might be expected to produce similar changes to barrier function in these structures. For example, the filtration rate in the kidney can be temporarily increased using the same technique, presumably through disruption of the “blood-urine barrier” [26].

In this study, we investigated in rats whether exposure by an ultrasound field in the presence of a circulating microbubble agent can induce a transient increase of retina vascular permeability for ocular drug delivery. MRI was used to guide the procedure, and the BRB disruption was verified through the delivery of an MRI contrast agent that normally does not reach the retina.

## Materials and Methods

### Animals

All experimental procedures were approved by the Animal Care Use Committee of Harvard medical school (Protocol 02674). Twenty male Sprague-Dawley rats (Charles River Laboratories, Boston, MA; weighing: 300–450 g) were used for this study. Sixteen animals were sonicated; four served as controls (Table 1). Before sonications, the animals were anesthetised with a mix of 80 mg/kg of ketamine (Aveco Co., Inc., Fort Dodge, IA) and 10 mg/kg of xylazine (Lloyd Laboratories, Shenandoah, IA) by IP injection. The hair around the eye was removed with clippers and depilatory cream (Nair, Church & Dwight Co., Inc., Princeton, NJ), and a catheter was placed in the tail vein. Body temperature was maintained throughout the procedure with a heated water pad.

**Table 1:** Summary of the experimental groups.

Pressures amplitude (MPa)	Number of rats/BRBD*	Number of rats for BRB recovery	Number of rats for histology
1.1	6/6	1^a^	1
0.88	4/4	0	1
0.81	6/6	3^b^	1
0 (Control)	4/0	0	1
Total rats	20/16	4	4

For blood-retinal barrier (BRB) recovery study, the second Gd-DPTA injection was administered 3.5h^a^ or 3h^b^ after the last sonication.

* BRBD: blood-retinal barrier disruption.

### Equipment

The ultrasound system and experimental setup were the same as used previously for BBB disruption in small animals [19]. The ultrasound field was generated using a custom-designed, single-element, spherically curved, air-backed transducer with a diameter of 100 mm and radius of curvature of 80 mm operating at a frequency of 690 kHz. The absolute and relative peak negative pressure amplitudes were measured in a water tank with a calibrated 0.5 mm diameter membrane hydrophone (Marconi, Chelmsford, UK). Reported exposure levels are peak rarefactional focal pressure (PRFP) amplitudes measured in water. The PRFP amplitude at the retina after transmission through the lens can be estimated by multiplying these values by a factor of 0.96, based on an attenuation coefficient and thickness of the lens of 1.4 dB/cm/MHz [27] and 3.5–4.0 mm, respectively. The attenuation of the other components of the rat eye (cornea, iris, vitreous) is negligible at this frequency. The pressure distribution of the transducer was mapped using a 0.2 mm needle hydrophone (Onda, Sunnyvale, CA) and a computer-controlled positioning system (step size: 0.25 mm). The half-maximum diameter and length of the focal pressure distribution were 2.3 and 12 mm, respectively.

**Figure 1:**
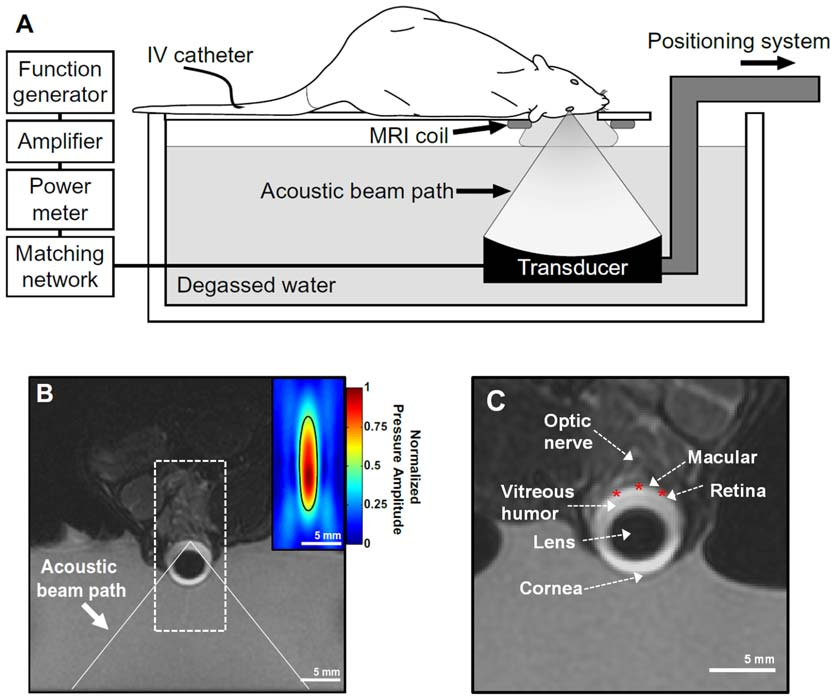
BRB disruption in the rat eye using an MRI-guided FUS system. (**A**) Schematic diagram of the experimental set up used to disrupt the BRB. (**B**) Coronal T2-weighted MR image of a rat eye within the sonication system. The eye was partially submerged in water to allow for acoustic coupling. The ultrasound beam path is superimposed. (**Inset**) Normalized focal pressure distribution of the focal region of the ultrasound beam (boxed region) displayed at the same scale as the MR image. (**C**) Relevant anatomical features of the eye visible in the MR image. The location of three of the five locations targeted for sonication are shown as star symbols.

The transducer was immersed in a tank of degassed water and mounted in a manually-operated, MR-compatible positioning system. Experiments were performed in a clinical 3 T MRI scanner (General Electric Healthcare, Milwaukee, WI). MRI was used for image guidance and evaluation of BRB disruption. Imaging was performed using a 7.5 cm diameter transmit/receive surface coil (constructed in-house). The experimental setup is diagrammed in Fig. 1A. Fig. 1B shows an MR image of the system with the ultrasound beam path superimposed. The inset in Fig. 1B shows the normalized pressure distribution in the focal region of ultrasound beam (boxed region drawn around the eye) at the same scale as the MR image. Relevant anatomical features in this image are noted in Fig. 1C.

The transducer was driven by a signal generated by an arbitrary waveform generator (Model 395, Wavetek Inc., San Diego, CA) and an RF amplifier (Model 240L, ENI Inc., Rochester, NY). The electrical impedance of the transducer was matched to the output impedance of the amplifier using an external inductor-capacitor tuning network. The electrical power was monitored with a power meter (Model E4419B, Agilent, Santa Clara, CA) and dual-directional coupler (Werlatone, Patterson, NY). The transducer efficiency was measured with a radiation force balance consisting of an absorbing brush attached to a digital scale.

### Sonications

Burst sonications (10 ms bursts applied at 1 Hz for 60 s) at PRFP amplitudes of 0.81, 0.88, and 1.1 MPa were delivered through the cornea and lens onto the retina in one eye of each rat, which laid in the lateral decubitus position on the sonication system (Fig. 1A). The eye was coupled to the tank containing the focused ultrasound (FUS) transducer via a bag of degassed water.

Five overlapping locations at the fundus of one eye in each animal were sonicated at the same PRFP amplitude. One target was centered on the optic nerve head; the others were at locations ±1.5 mm away in the left/right and anterior/posterior directions. Three of the target locations are indicated by star symbols in Fig. 1C. Each sonication was applied 10 s after an IV bolus injection of ultrasound contrast agent (USCA) microbubble suspension (Definity, Lantheus Medical Imaging, N. Billerica, MA). Immediately after “activation” of this agent, the suspension contains approximately 1.2×10^10^ lipid-shelled microbubbles/mL with mean diameter range of 1.1 µm–3.3 µm. For the current study, the solution was diluted 10 times in PBS and was injected IV at dosage of 20 µl/kg, which is approximately double the recommended dose for clinical use with ultrasound imaging. The administration of Definity was followed by an injection of 0.2 mL normal saline (0.9% NaCl) to flush the agent from the catheter in the tail vein. Sonications of the individual locations were spaced at least 2 min apart to allow the agent to mostly clear from the blood vasculature.

### Magnetic resonance imaging

MRI procedures were similar to those described previously for BBB investigations in small animals [19]. Before the rat experiments, the target location of the FUS beam in the MRI coordinate-space was visualized by imaging temperature changes induced in a silicone phantom with a T1-weighted fast spin echo (FSE) sequence. Then, the animal was placed on the sonication system. T2-weighted FSE images (Repetition time (TR): 2000 ms, echo time (TE): 85 ms, echo train length (ETL): 8, number of excitations/averages (NEX): 2) was used to select the targets. After sonication, serial contrast- enhanced T1-weighted FSE images (TR/TE: 500/17 ms, ETL: 4, NEX: 4) were acquired every 5 min up to 30 min to visualize the BRB disruption. These images were acquired before and after an IV injection of the MRI contrast agent Gadopentetate dimeglumine (Gd-DTPA) (Magnevist, Berlex Laboratories, Inc., Wayne, NJ, USA; molecular weight: 938 Da) administered as a bolus at a dose of 0.125 mmol/kg. In four animals, the contrast-enhanced imaging was repeated with the second Gd-DTPA injection 3 or 3.5 h after sonication (Table 1). The T2- and T1-weighted FSE images were obtained with an 8 cm field of view, a matrix size of 256×256, and a slice thickness of 1 mm.

### Histology

The animals were sacrificed 24 h after the last sonication. Each animal was deeply anesthetized, sacrificed, and its eye fixed via transcardial perfusion (0.9% NaCl, 100 mL; 10% buffered formalin phosphate, 250 mL). A representative example from each experimental group (Table 1) was examined in light microscopy. For this examination, the eye was embedded in paraffin and serially sectioned at 5 µm in the coronal plane (parallel to the direction of ultrasound beam propagation). Every 50^th^ section (250 µm apart) was stained with haematoxylin and eosin (H&E). Three sonicated and one non-sonicated (control) eyes were examined. The author (NV) who evaluated the histology was blind to the FUS exposure parameters.

### Data analysis

MR image analysis was performed in MATLAB (The MathWorks, Natick, MA, USA). A region of interest (ROI) was selected in the contrast-enhanced T1-weighted image that included the sonicated area of the retina and the vitreous humor. The mean signal intensity in the ROI was found as a function of time for the time-series of images. The percent signal intensity enhancement was found by normalizing the measurements to those made in an image obtained before Gd-DTPA administration. The enhancement in the sonicated areas was compared to that measurements obtained in a similar region of interest selected in control (non-sonicated) animals using an unpaired two-tailed t-test. Values of *p*<0.05 were considered statistically significant.

## Results

### MRI analysis of BRB disruption

**Figure 2:**
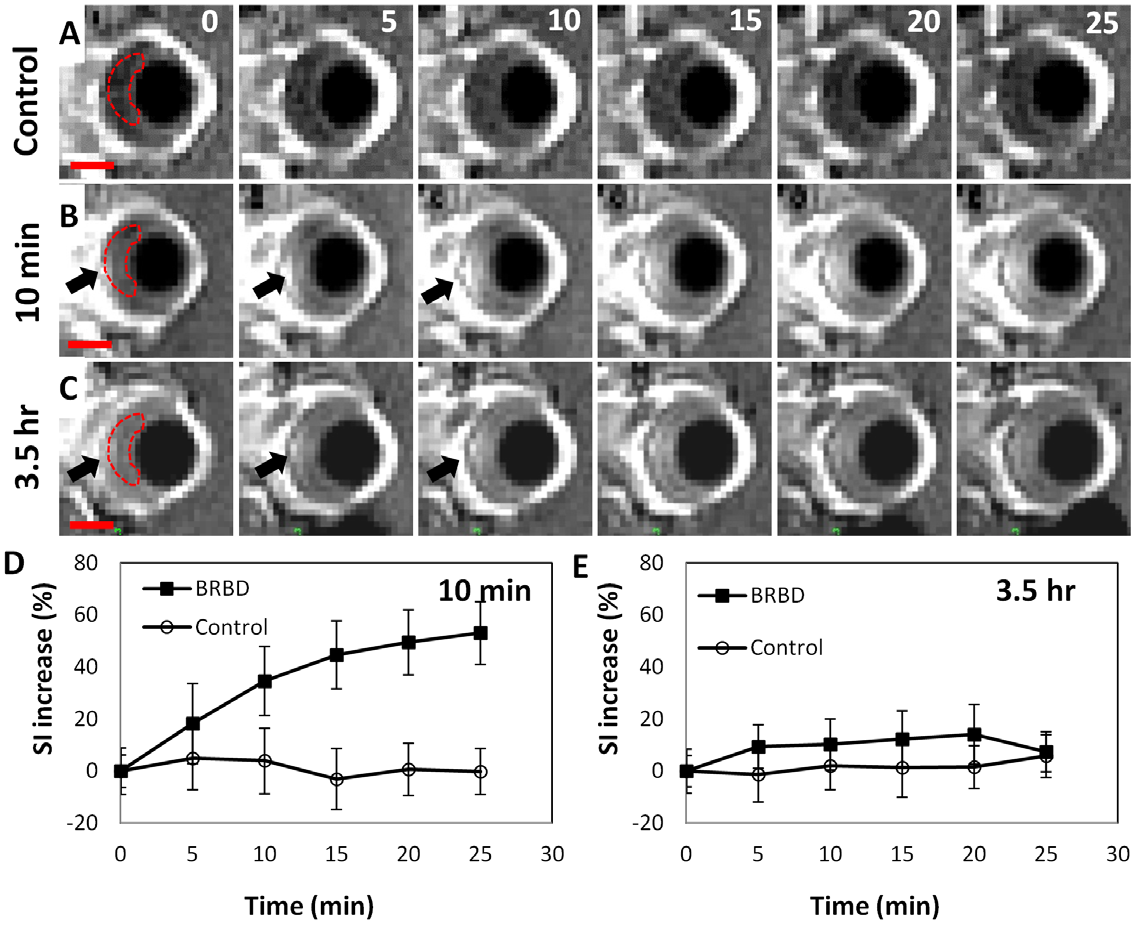
BRB disruption in the rat eye after sonication with microbubbles at five overlapping targets centered on the optic nerve head. (**A–C**) Time-series of sagittal contrast-enhanced T1-weighted images of a rat eye acquired at 5 min intervals after an IV injection of MRI contrast agent (Gd-DTPA). (**A**) Control eye. (**B**) Gd-DTPA injection 10 min after the last sonication. (**C**) The second Gd-DTPA injection 3.5 h after the sonications to examine the recovery of the BRB. (**D–E**) The normalized signal intensity (SI) increase (spatial averaged mean ± S.D.) due to extravasation of Gd-DTPA was measured as a function of time in a region of interest (dotted line) that included the sonicated retina and the vitreous. In **C** and **E**, the BRB was not fully recovered at 3.5 h. The ultrasound beam propagated from right to left with respect to these images. The peak rarefactional pressure amplitude for sonications was 1.1 MPa with 20 µl/kg microbubbles USCA (Definity). Scale bars in **A–C:** 2 mm.

In each animal, a time-series of contrast-enhanced T1-weighted images of the eye was obtained to evaluate the integrity of the BRB (Fig. 2). No signal enhancement suggesting any Gd-DTPA leakage into the retina or vitreous humor was observed in non-sonicated (control) animals. Only those animals that received FUS and microbubbles resulted in detectable signal enhancement.

Figure 2 shows the results of a typical experiment with the highest FUS pressure amplitude tested (1.1 MPa). The BRB disruption was observed initially as signal enhancement in the retina at the sonicated locations (arrows in Fig. 2B). At later times enhancement was observed in the vitreous humor as the Gd-DTPA diffused out from the retina. Ultimately the signal in entire vitreous humor appeared to be enhanced. The mean signal intensity at the ROI indicated by the dotted line in Fig. 2B increased by over 50% compared to the baseline, while the signal intensity increase at control eye was near zero (Fig. 2D). The sonicated eye was imaged again 3.5 h following the last sonication with the second MRI contrast injection (Fig. 2C). Low-level BRB disruption was still observed but the level was substantially less compared to the first MRI contrast injection. The maximum signal intensity enhancement at 3.5 h was only around 15% compared to the baseline (Fig. 2E).

**Figure 3:**
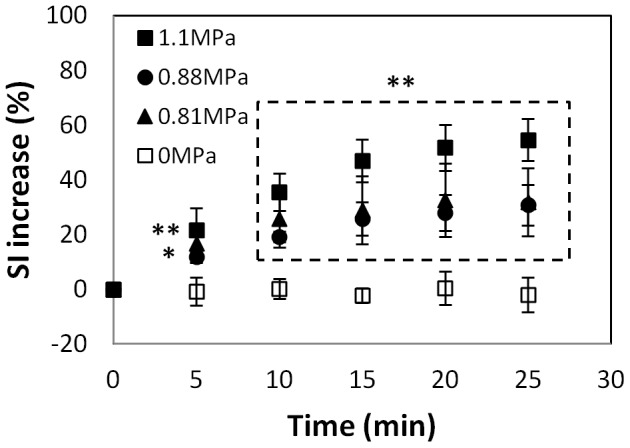
The average normalized signal intensity (SI) change measured in a time-series of contrast enhanced T1-weighted images for all animals (mean ± S.D.). The horizontal axis shows the interval after Gd-DTPA injection, which was administered 10 min after the last sonication in each eye. The measurements were obtained in a region of interest that included the sonicated portion of the retina and the vitreous humor. The pressure amplitudes of these sonications were: 0.81 (N = 6), 0.88 (N = 4), 1.1 MPa (N = 6) for sonicated eyes and 0 MPa (n = 4) for the control eyes. The SI increase in the sonicated eyes was significantly larger than that in the controls at every time point (* *p*<0.05; ** *p*<0.01).

**Figure 4:**
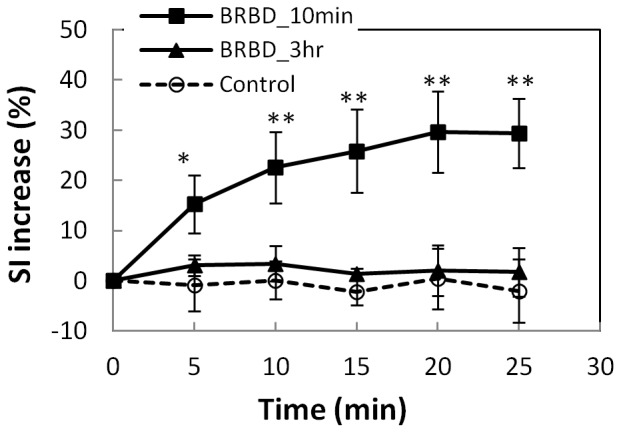
Recovery of the BRB 3 h after sonication at 0.81 MPa. The signal intensity (SI) increase measured in a ROI that included the retina and the vitreous humor is plotted as a function of time after two injections of MR contrast agent (Gd-DTPA). After the first injection (10 min after the last sonication), the SI increase was significantly larger (* *p*<0.05; ** *p*<0.01) than the controls for every point in the time-series. After the second injection (3 h after sonication), the SI changes were not significantly different than the controls (*p*>0.05) at any time point. Data shown are the average SI increase (mean ± S.D.) for three sonicated eyes and four controls.

MRI contrast enhancement in the retina and vitreous humor similar to that seen in Fig. 2 was observed after FUS at the three PRFP amplitude tested: 0.81 (N = 6), 0.88 (N = 4), and 1.1 MPa (N = 6), in each of the sonicated eyes (N = 16) and in none of the controls (N = 4). The maximum signal enhancement increase was the largest after sonication at 1.1 MPa. Sonications at 0.81 and 0.88 MPa produced similar levels of enhancement (Fig. 3). Signal intensities at all FUS exposure groups were significantly higher than those at control group at each time point (*p*<0.05). At the lowest pressure amplitude tested (0.81 MPa), the BRB appeared to be restored in three animals imaged 3 h after sonication with the second injection of MRI contrast agent (Fig. 4). In these animals, a mean enhancement level of 30% above baseline was achieved after the first Gd-DTPA injection was administered. After the second injection 3 h later, the mean enhancement level was less than 5% above the baseline and was not significantly different (*p*>0.05) compared to what was achieved in the control animals.

### Histology analysis

**Figure 5:**
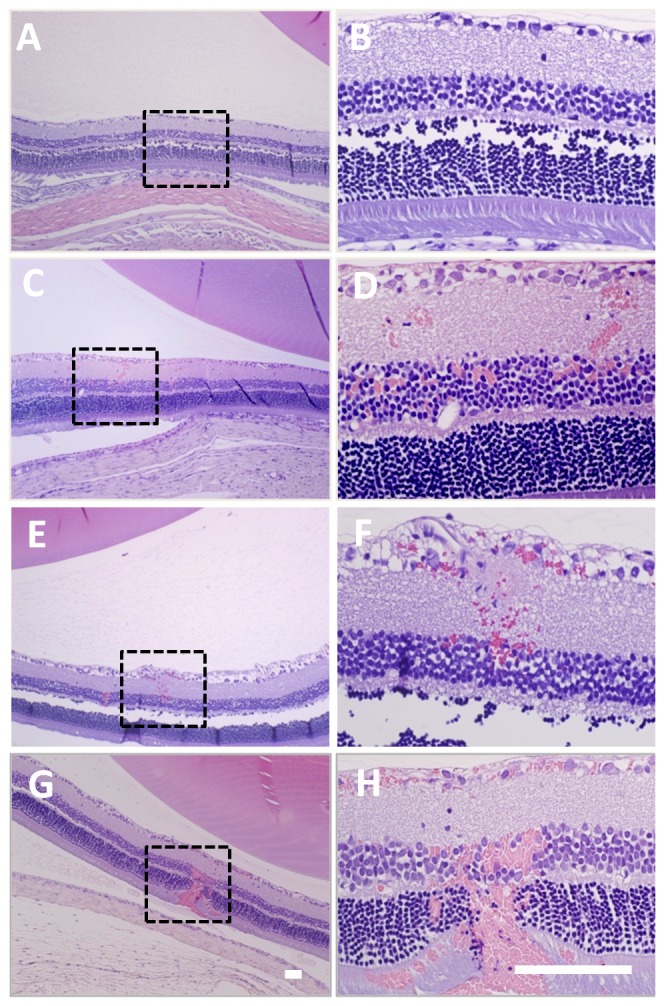
Microphotographs of H&E-stained sections from representative examples from each experimental group (**Table 1**). Images on the right are high-magnification views of the boxed regions shown on the left. (**A–B**) Non-sonicated (control) eye. (**C–H**) The largest effects found on histological examination in each BRB disrupted eye at increasing acoustical pressure amplitudes: (**C–D**) 0.81 MPa; (**E–F**) 0.88 MPa; (**G–H**) 1.1 MPa. At 0.81–0.88 MPa, the retina and other ocular structures appeared mostly normal except for tiny petechaie found in the nuclear layers of the retina. At 1.1 MPa, more extensive petechaie were found. The animals were sacrificed 24 h after the sonications. The retinal separations evident in these images were artifacts that occurred during formalin fixation and tissue preparation. Scale bars: 100 µm.

Histological examination was performed on three sonicated eyes which showed the largest effect on contrast enhanced T1-weighted MRI; one for each of the three pressure amplitudes tested and one control eye. At 0.81 and 0.88 MPa, the retina in the sonicated region appeared to be generally unaffected in the H&E-stained sections except for few tiny (∼100 µm) clusters of extravasated erythrocytes (petechaie) found in the nuclear layers of the retina. Other structures that were exposed to FUS along the beam path (choroid, cornea, iris, lens, optic nerve) appeared normal. More extensive damage such as more petechaie and retinitis were observed after sonication at 1.1 MPa. Figures 5 shows retina from the control eye (Fig. 5 A–B) and the largest effects found in each sonicated eye on histological examination (Fig. 5 C–H).

## Discussion

This study demonstrated that appropriately powered FUS combined with circulating microbubbles can induce a transient and reversible disruption of the BRB, resulting in extravasation of a systemically injected MRI contrast agent into the retina and vitreous. By combining the BRB disruption and systemic injection, this method could be used to deliver therapeutic agents or imaging probes which normally do not enter the retina. The BRB appeared to be restored within a few hours, which could provide a good time-window for ocular drug delivery while avoiding unwanted effects that may result from long-term BRB disruption. Gd-DTPA was observed to leak into the vitreous humor after sonication, and over 25 min, this enhancement covered its entire extent within the vitreous humor. Any agent that leaks out of the retinal blood vessels using this technology would thus potentially be delivered to the entire retina. Presumably the retinal tissue where the BRB was disrupted would receive a higher dose than regions that receive drug via the vitreous, but this would need to be verified.

A number of methods have been evaluated to overcome the BBB for drug delivery, including intraarterial infusion of a hyperosmotic solution such as mannitol to diffusely disrupt the barrier, or modifying or encapsulating drugs to enable their transport across the brain endothelium [28]. In principle, similar techniques could be used to deliver drugs to the retina [29]. However, those methods would be either invasive, non-targeted, or require the development of new drug formulations, which may change the drug's pharmacokinetic properties. Such strategies may also result in the delivery of agents to the CNS. In contrast, FUS combined with microbubble is noninvasive, localized, repeatable, and could utilize already developed pharmacological agents without modification of drug properties.

FUS devices designed to target the retina for drug delivery would be relatively simple to construct compared to other targets (particularly the brain), due to the eye's superficial location. The therapeutic use of FUS in the eye has a long history, with prior studies investigating it for a variety of applications including treatments for glaucoma and ocular tumors and for transcorneal drug delivery [30], [31], [32], [33], [34], [35], [36], [37], [38], [39]. In fact, the first FDA-approved high-intensity focused ultrasound device was for applications in the eye [40]. This prior experience can be useful the development of devices for use in humans and for clinical translation.

There is less prior experience with microbubble-enhanced ultrasound in the eye. While investigators have utilized contrast-enhanced ultrasonography in the eye [41], [42], and others have injected microbubbles into the vitreous along with viral vectors or siRNA for retinal delivery via sonoporation [43], [44], we are not aware of prior work with circulating microbubbles and sonication parameters similar to those used in the present study. One study by Hirokawa et al. [45] investigated bioeffects produced in the retina after ultrasound imaging with a microbubble USCA using fundus angiography. In that work, which used a 2 MHz transducer to sonicate the rabbit eye, increased retinal permeability was noted in one of five animals (peak rarefactional focal pressure (PRFP): 2.0 MPa), and vasoconstriction was noted in four of five cases. In our current study, we achieved an increase in retinal permeability at substantially lower PRFP amplitudes (0.81–1.1 MPa). While it is difficult to directly compare our findings to that work because of the multitude of differences in the exposure conditions (frequency, burst length, pulse repetition frequency, USCA dose, etc.), the disparate outcomes are generally consistent with what has been observed in the brain. Experiments testing brain sonications have found that higher pressure amplitude is needed to induce BBB disruption when the ultrasonic frequency is increased [46] and when the burst length decreased [47]. Others have also shown that vasoconstriction can occur during sonication in the brain with FUS and microbubbles [48]. Thus, while more work is needed to evaluate the bioeffects observed in the retina after sonication with microbubbles, we might expect that prior results achieved in the brain may point to how BRB disruption can be optimized.

If the vascular response to FUS and microbubbles in the retina and the brain are indeed similar, we might expect that BRB disruption could be achieved at lower amplitudes than were tested here. Previous work in rabbits has shown that the threshold for BBB disruption at 690 kHz is between 0.3–0.4 MPa [46], and that significant petechaie and mild parenchymal damage occurs at 0.8 MPa [19]. Here, we used higher PRFP amplitudes (estimated to be 0.78–1.06 MPa at the retina), double the USCA dose, and a twice the sonication duration than was used in the brain studies [46]. These parameters were used to achieve signal intensity increases in contrast MRI similar to what has been observed in the brain.

Detecting MRI contrast at a lower level with our MRI protocol may be challenging. The vascular density in the retina is lower than in the brain, and the amount of tracer that leaks into the extravascular space will be smaller. Furthermore, the retinal thickness in the rat (∼200 µm) is a small fraction of the voxel dimensions in our MR imaging, which will lead to volume-averaging and reduced sensitivity to low-level BRB disruption. While volume averaging errors will be reduced as Gd-DTPA diffuses into the vitreous, the concentration in the vitreous may be small for low-level BRB disruption. Future work should test more sensitive methods to investigate the threshold for BRB disruption, such as optical techniques using fluorescent tracers or high-field MRI using a small eye coil.

Being able to achieve BRB disruption at lower exposure levels may reduce the risk for side effects. While we did not see retinal damage at the two lower exposure levels (0.81 and 0.88 MPa), we did find tiny petechaie at a few locations in each eye. We sonicated five locations in each eye; most of the retina was sonicated and demonstrated BRB disruption. The fact that petechaie were detected in only few focal areas (Fig. 5) suggests that perhaps we were operating just above the threshold for inertial cavitation and that BRB disruption without these petechaie could be achieved at lower PRFP amplitudes than were used here.

The risk for side effects may also be reduced using a transducer that produces a smaller focal region, which could be achieved using a higher ultrasound frequency. We used a low frequency (690 kHz) for this initial work based on parameters that we have been using for BBB disruption. In the brain, use of a lower frequency appears to pose less risk for petechaie than higher values closer to the resonant frequency of the microbubbles [46]. A lower frequency also will be less affected by the lens, which may distort the ultrasound field. However, low frequency ultrasound may not be ideal for retinal sonications due to the size of the focal region and the potential for unwanted effects to surrounding tissues. Future work is needed to optimize the FUS device and the sonication parameters for this application and to evaluate the sonication effects. In particular, it may be desirable to avoid sonication through the lens. For humans and other animals with larger eyes, this could be achieved using a transducer with a toroidal geometry.

### Outlook

This feasibility study was limited, and additional future work on this technique is needed to advance this technique. We only evaluated a relatively narrow range of exposure conditions, and our sonication system and imaging were not optimized. A broader range of parameters should be evaluated with devices designed for ocular sonication to optimize the exposures and minimize the risk for side effects. We likely had some variation from target-to-target in the actual PRFP amplitude at the retina, as we did not take into account beam aberration induced by the lens, clipping of the FUS beam by the bone in the eye socket, and reflections from the bone behind the eye. More reliable exposures might be achieved by recording acoustic emissions to evaluate the microbubble dynamics and control the sonication system [49] and by integrating a fundus camera into the FUS system to monitor the retina in real-time. We also only evaluated short-term histological effects of the sonications, and future work should evaluate whether the procedure induces any functional changes. For example, electroretinograms (ERG) can be performed after the sonications to evaluate the effects on retinal function. Finally, the safety of delivering therapeutic agents to the retina also needs to be evaluated. Delivery of therapeutic agents such as anti-VEGF antibodies, genes, or siRNA for degenerative diseases such as age-related macular degeneration could be applications with a good opportunity to improve therapeutic outcomes.

Despite the limitations of this study, these results are a major step forward in producing a noninvasive alternative to ocular drug delivery for treatment of retinal disorders such as age-related macular degeneration, diabetic retinopathy and retinitis pigmentosa. Retinal tumors may also benefit from this approach through the delivery of agents to infiltrating cancer cells that are protected by the BRB and by increasing drug delivery to the vascular part of the tumor. In addition, this technology would provide an alternative for patients who cannot tolerate intravitreal injection due to a risk of retinal detachment or increased intraocular pressure.

### Conclusion

In conclusion, these experiments have shown that FUS and microbubbles can induce a temporary disruption of the BRB in rats. Using FUS exposure parameters similar to those used previously to disrupt the BBB, we found that we could deliver an MRI contrast agent that normally does not extravasate to the retina and the vitreous humor due to the presence of the BRB. No significant retinal damage was found in histology at the two lower acoustic pressure amplitudes tested, and the barrier was found to be restored 3 h after sonication. This technique could provide a noninvasive method to deliver drugs to the retina, avoiding the need for intraocular injections.
